# Prognostic value of serum leptin in advanced lung adenocarcinoma patients with cisplatin/pemetrexed chemotherapy

**DOI:** 10.3892/ol.2014.1988

**Published:** 2014-03-21

**Authors:** WENJUN MOU, HUI XUE, HONGLI TONG, SHENGJIE SUN, ZHUHONG ZHANG, CHUNYAN ZHANG, QIYU SUN, JING DONG, XINYU WEN, GUANGTAO YAN, YAPING TIAN

**Affiliations:** 1 School of Medicine, Nankai University, Tianjin 300071, P.R. China; 2 Department of Clinical Biochemistry, Chinese PLA General Hospital, Beijing 100853, P.R. China; 3 Research Laboratory of Biochemistry, Basic Medical Institute, Beijing 100853, P.R. China; 4 Oncology Department, Chinese PLA General Hospital, Beijing 100853, P.R. China

**Keywords:** leptin, lung adenocarcinoma, cisplatin/pemetrexed, prognosis

## Abstract

Cisplatin/pemetrexed chemotherapy has been established as a standard treatment in lung adenocarcinoma. However, the response to the cisplatin/pemetrexed combination varies considerably among patients due to individual variations. Thus, novel biomarkers are required to aid the prediction of the response to the cisplatin/pemetrexed combination. We hypothesized that leptin expression may be a determinant for prognosis in lung adenocarcinoma patients with cisplatin/pemetrexed chemotherapy. Serum from consenting patients with lung adenocarcinoma were obtained for the measurement of leptin and associated tumor biomarkers. Leptin expression was measured by radioimmunoassay. Carcinoembryonic antigen (CEA), carbohydrate antigen 19-9 (CA19-9), CA15-3, CA125, CA72-4, cytokeratin 19 fragment (CYFRA21-1) and neuron-specific enolase (NSE) expression were determined by electrochemiluminescence immunoassays. Serum squamous cell carcinoma antigen levels were measured using a microparticle enzyme immunoassay. The associations between serum leptin and tumor biomarker expression were evaluated by Spearman’s correlation analysis. Serum CEA, CA19-9, CA15-3, CA125, CA72-4, CYFRA21-1 and NSE levels showed no obvious difference among patients. However, a trend towards an improved prognosis was observed in patients with lower serum leptin at diagnosis and an increase during cisplatin/pemetrexed chemotherapy. The results indicated that the serum leptin level has prognostic indications in patients with advanced lung adenocarcinoma during cisplatin/pemetrexed chemotherapy, which indicates that it may be a useful marker for the prognosis of cancer patients undergoing chemotherapy treatment.

## Introduction

Lung cancer is the main cause of cancer-related mortality in Eastern Asia and worldwide (1). Lung adenocarcinoma accounts for ~70% of non-small cell lung cancer and is now becoming more frequently detected (2). Recent studies have demonstrated that lung adenocarcinoma is a genetically and clinically heterogeneous tumor with malignant behaviors (2,3).

For patients with advanced lung adenocarcinoma, treatment with pemetrexed in combination with cisplatin is recommended for the first-line therapy (4). However, the response to cisplatin/pemetrexed treatment varied among patients (4,5). Certain patients benefit from the cisplatin/pemetrexed regimen, whereas others react with no remission or progress rapidly. The distinct clinical outcome may be largely dependent on the individual variation (3,6). In this regard, the prospective identification of those patients who are mostly likely to respond to cisplatin/pemetrexed using prognostic markers are required.

Evidence from clinical studies has shown a strong association between the cytokeratin 19 fragment (CYFRA21-1) and its prognostic value in lung adenocarcinoma. Park *et al* (7) showed that high preoperative CYFRA21-1 may be a determinant for a poor prognosis in patients with lung adenocarcinoma who have undergone surgery. By contrast, Ono *et al* also identified that the serum levels of CYFRA21-1 may also possess prognostic indications for patients diagnosed with advanced lung adenocarcinoma (8). Other studies have also shown the prognostic value of squamous cell carcinoma (SCC) antigen (9–11), carcinoembryonic antigen (CEA) (11–13), carbohydrate antigen 125 (CA125) (11,14) and even neuron-specific enolase (NSE) (15) in non-small cell lung cancer. It has also been shown that the most effective way of evaluating prognosis is dynamically, by comparing pre- and post-therapy results (16,17).

Leptin, mainly synthesized by adipocytes (18), is a pleiotropic molecule that is involved in tumorigenesis and the progression of lung cancer (19–21). Recent evidence indicates that leptin shows a prognostic value for the overall survival rate in patients with gastric adenocarcinoma (22), hepatocellular carcinoma (23) and colorectal adenocarcinoma (24).

The aim of the present study was to evaluate whether serum levels of leptin, CEA, CA19-9, CA15-3, CA125, CA72-4, CYFRA21-1, NSE and SCC have prognostic indications in patients with lung adenocarcinoma who are treated with cisplatin/pemetrexed chemotherapy in the first-line setting.

## Materials and methods

### Patients and treatment

A total of 49 previously untreated patients with histological evidence of primary lung adenocarcinoma or mucous adenocarcinoma who were admitted to the Oncology Department of the Chinese PLA General Hospital (Beijing, China) in 2012 were prospectively enrolled. The study was approved by the ethics committee of the Chinese PLA General Hospital. All patients provided written informed consent. The following variables were recorded: Age, gender, body mass index, tumor histology, Eastern Cooperative Oncology Group (ECOG) performance status and other associated clinical parameters. All patients were treated with first-line cisplatin/pemetrexed chemotherapy. Only 27 patients who received at least four cycles of first-line treatment with the cisplatin/pemetrexed chemotherapy were eligible. A total of 22 individuals who refused or reported inability to collaborate were excluded. Computed tomography was performed to assess tumor response by the Response Evaluation Criteria in Solid Tumors criteria, version 1.1 (25).

### Measurement of serum leptin and routine tumor biomarker levels

Serum samples were collected within 2 h and stored at −80°C. Tumor biomarkers, including CEA, CA19-9, CA15-3, CA125, CA72-4, CYFRA21-1 and NSE, were measured using electrochemiluminescence immunoassays (E170; Roche Diagnostics, Basel, Switzerland). Serum SCC was measured using a microparticle enzyme immunoassay (i2000; Abbott Diagnostics, Lake Forest, IL, USA). All tests were performed according to the manufacturer’s instructions. The cut-off points for each tumor biomarker, determined by the manufacturer, were as follows: CEA, 5 μg/l; CA19-9, 37 U/ml; CA15-3, 30 U/ml; CA125, 35 U/ml; CA72-4, 10 U/ml; CYFRA21-1, 4 ng/ml; NSE, 24 ng/ml; and SCC, 1.5 ng/ml.

The serum leptin levels of 27 patients and age- and gender-matched healthy controls were measured using radioimmunoassay kits as previously described (26).

### Statistical analyses

Statistical analyses were carried out using SPSS 16.0 (SPSS, Inc., Chicago, IL, USA). Data are presented as the mean, median and range. The difference of serum leptin levels among the groups were examined using one-way analysis of variance. Spearman’s co-efficient was used for correlations of the associated quantitative data. P<0.05 was considered to indicate a statistically significant difference.

## Results

### Patient characteristics

The patient characteristics are presented in Table I. The majority of patients were elderly and predominately male (mean age, 50 years; range, 30–69 years; female to male ratio, 7:20). All 27 patients were diagnosed with adenocarcinoma. A total of 21 patients (77.8%) were classified as stage IV, four patients (14.8%) as stage IIIB and the remaining two patients (7.4%) with an unknown stage. All patients had a good ECOG performance; 17 patients with ECOG=0 and 10 with ECOG=1. Only two patients were diagnosed with weight loss.

### Association between routine clinical tumor markers and its prognostic value in lung adenocarcinoma in response to cisplatin/pemetrexed chemotherapy

To identify the association between routine clinical tumor markers and its prognostic value, the patients were divided into three groups according to their response to the cisplatin/pemetrexed chemotherapy treatment. Among these 27 patients, nine retained stable disease (SD) following four cycles of cisplatin/pemetrexed, whereas nine had a partial response (PR) and nine had progressive disease (PD). All of them were confirmed by clinical and radiological assessment. The routine serum tumor biomarker levels were analyzed at diagnosis in the PR, SD and PD subgroups. All tumor biomarkers, including CEA, CA19-9, CA15-3, CA125, CA72-4, CYFRA21-1 and NSE, showed no clear difference among the PR, SD and PD subgroups; Table II.

### Association between serum leptin and its prognostic value in lung adenocarcinoma in response to cisplatin/pemetrexed chemotherapy

**Figure 1:**
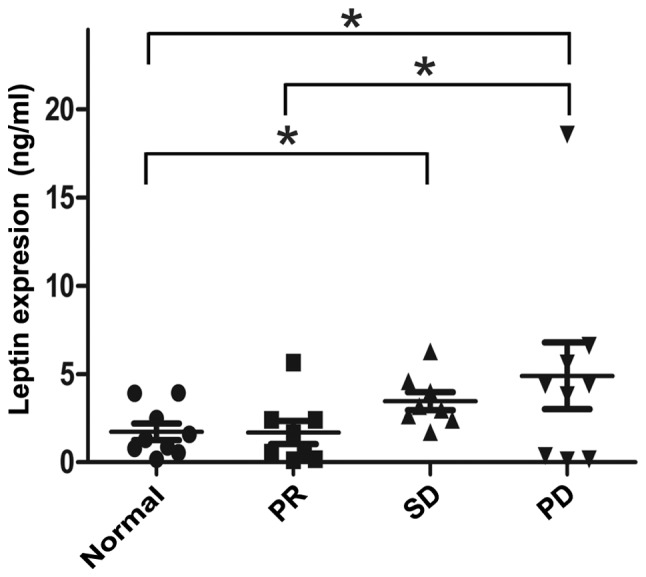
Comparison of serum leptin levels in the PR, SD and PD subgroups with various responses to cisplatin/pemetrexed chemotherapy. Each symbol represents one individual and horizontal lines represent median values. PR, partial response; SD, stable disease; PD, progressive disease. P=0.021, normal vs. PD; P=0.041, PR vs. PD; and P=0.023, normal vs. SD.

Serum leptin levels at diagnosis were determined by a radioimmunoassay. A statistically significant difference was observed among the PR, SD and PD subgroups; Fig. 1. The serum leptin levels at diagnosis were significantly higher in the lung adenocarcinoma patients showing PD compared with patients in the PR subgroup; 6.19 (0.36–18.57) versus 1.69 (0.12–5.64) ng/ml, P=0.021; Fig. 1. The serum leptin levels of the healthy controls and patients with PD were almost the same; 1.73 (0.18–3.93) versus 1.69 (0.12–5.64) ng/ml. Fig. 1 also shows significant elevated serum leptin levels in patients with PD compared with controls; 6.19 (0.36–18.57) versus 1.73 (0.18–3.93) ng/ml, P=0.009. In addition, the leptin levels were higher in the patients with SD compared with controls; 3.47 (1.71–6.27) versus 1.73 (0.18–3.93) ng/ml, P=0.013.

To demonstrate the correlation between the leptin levels and routine clinical tumor markers in lung adenocarcinoma in response to cisplatin/pemetrexed chemotherapy, Spearman’s correlation was performed between the serum leptin levels and the routine tumor biomarkers, including CEA, CA125, CA15-3, CA19-9, CA72-4, CYFRA21-1, NSE and SCC. Regarding the assessed parameters (Table III), only serum SCC showed a direct correlation in association with serum leptin levels; r=−0.398 and P=0.012. No statistically significant associations between serum leptin and other routine biological parameters were found.

**Figure 2:**
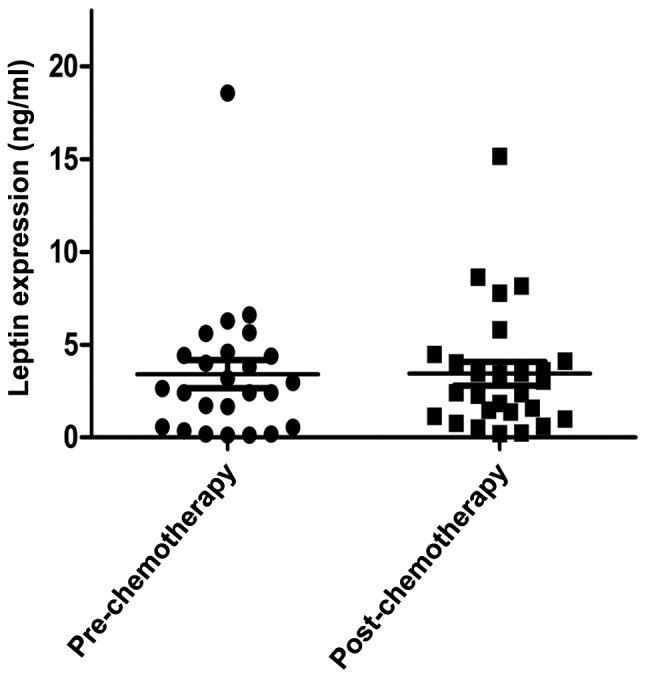
Comparison of serum leptin levels pre- and post-cisplatin/pemetrexed chemotherapy in advanced lung adenocarcinoma patients. Each symbol represents one individual and horizontal lines represent median values.

**Figure 3:**
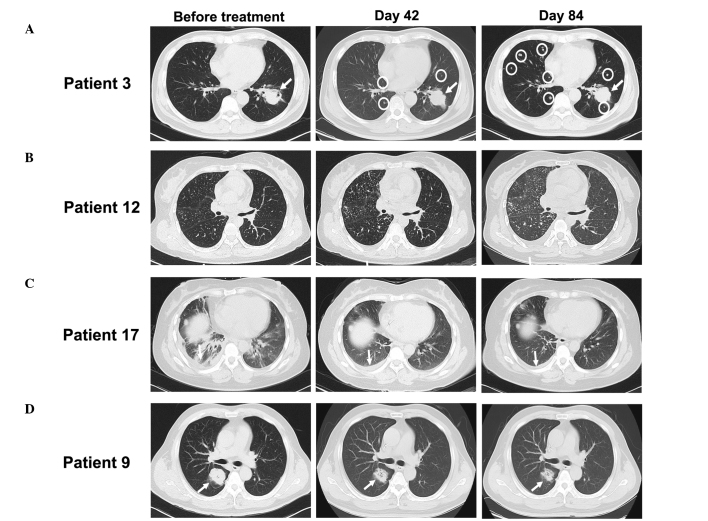
Computed tomography of patients with various responses to cisplatin/pemetrexed chemotherapy. (A) PD in a patient who progressed with an obvious increase in the volume of pulmonary nodules (white arrows) and more emerging micro-lung metastases (white circles). (B) SD in a patient with more emerging micro-lung metastases and pleural effusion (arrows). (C) SD with less pleural effusion (arrows). (D) PR in a patient. The arrows indicate an obvious regression in the volume of pulmonary nodules. Day 42 and day 84 indicate two and four cycles of cisplatin/pemetrexed respectively. PD, progressive disease; SD, stable disease; PR, partial response.

**Figure 4:**
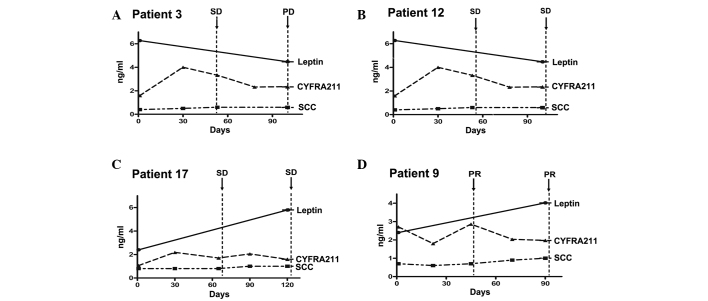
Change of serum leptin, SCC and CYFRA21-1 in patients with various responses during cisplatin/pemetrexed chemotherapy. (A, B, C and D) Serum leptin levels for four patients (one per panel) across multiple time points. The status of disease at various times is shown and the arrows represent the timing of computed tomography assessment. SCC, squamous cell carcinoma; CYFRA21-1, cytokeratin 19 fragment; SD, stable disease; PD, progressive disease; PR, partial response.

Furthermore, the change of serum leptin levels were evaluated during chemotherapy. There was no statistically significant difference pre- and post-chemotherapy; Fig. 2. However, when the patients were divided into PR, SD and PD subgroups, it was found that the change of serum leptin during cisplatin/pemetrexed chemotherapy corresponded to chemotherapy responses. The application of cisplatin/pemetrexed chemotherapy provided a significant decrease in serum leptin levels in eight out of nine patients with PD (Figs. 3A and 4A). The serum leptin levels increased in seven of nine patients with PR (Figs. 3D and 4D). Regarding the patients with SD, patients with exacerbated tumor burden (5/9) showed an obvious reduction in serum leptin levels (Fig. 3B and 4B), while patients in remission (4/9) showed an increase in serum leptin (Figs. 3C and 4C).

The data comparing the serum leptin, SCC and CYFRA21-1 values were also available across four serial time points during chemotherapy for all 27 patients. The serum SCC levels were slightly elevated at one or more time points in almost all patients following cisplatin/pemetrexed chemotherapy; Fig. 3. CYFA21-1 exhibited no regular expression pattern, corresponding to responses to cisplatin/pemetrexed during chemotherapy; Fig. 3.

In conclusion, a trend towards an improved prognosis was observed in patients with lower serum leptin levels at diagnosis and an increase during cisplatin/pemetrexed chemotherapy.

## Discussion

In the present study, the serum leptin level was demonstrated in patients with lung adenocarcinoma to show a trend that corresponded to the cisplatin/pemetrexed chemotherapy response. This is the first study, to the best of our knowledge, that demonstrates that serum leptin has prognostic indications in patients with lung adenocarcinoma who are treated with cisplatin/pemetrexed chemotherapy in the first-line setting.

Cisplatin/pemetrexed has a well-established clinical activity in lung adenocarcinoma (27). Clinically, the majority of oncologists consider cisplatin/pemetrexed as a standard chemotherapy regimen for patients with lung adenocarcinoma. Several studies have demonstrated the association between the ERCC1, RRM1 and CHFR genes (28–30) and its prognostic value in response to platinum compounds, gemcitabine and taxanes. However, the best markers in predicting the response to cisplatin/pemetrexed treatment remain a matter of debate. The results of the present study showed that the level of serum leptin has a prognostic value for differentiating the treatment benefit of cisplatin/pemetrexed. Lower leptin levels at diagnosis were followed by PR, while higher leptin levels were followed by PD. This indicated that the serum leptin expression may be associated with the response to cisplatin/pemetrexed. Previous studies are consistent with the prognostic value of serum leptin for overall survival rates in patients with gastric adenocarcinoma, hepatocellular carcinoma and colorectal adenocarcinoma (22–24). The results in our studies revealed the potential role of serum leptin as a prognostic marker in response to the cisplatin/pemetrexed chemotherapy. This finding would be of great significance for the adjustment of the chemotherapy regimen according to the serum leptin level in the near future.

Notably, the results of the present study showed a significant change of serum leptin that corresponded with chemotherapy responses. Inconsistent with this, a study by Tas *et al* (19) indicated that the serum leptin level was decreased owing to the chemotherapy effect. Although the numbers of patients with varied responses were limited in the present study, a significant decrease in serum leptin levels were observed in eight out of nine patients with PD. Serum leptin was clearly elevated in seven out of nine patients with PR. However, the reduction of leptin was regardless of the response to chemotherapy and did not reach a statistical significance (P=0.06) in the study by Tas *et al* (19). In addition, the studies by Tas *et al* (19) and Kerenidi *et al* (31) showed that serum leptin levels in lung cancer patients were lower when compared with healthy individuals. The results of these studies are inconsistent with the present study and may in part reflect the various populations tested and the inherent variability in quantifying serum leptin using ELISA and radioimmunoassay.

In conclusion, the results of the present study indicate a strong association between serum leptin level and its prognostic value in patients with advanced lung adenocarcinoma during cisplatin/pemetrexed chemotherapy. Considering the small number of patients, future studies with pro rata patients are required to further confirm the prognostic value of serum leptin. The findings of the present study will aid the understanding of the mechanism of patient response to the cisplatin/pemetrexed combination and aid the development of a novel prognostic marker.

## Figures and Tables

**Table I:** Characteristics of the 27 lung adenocarcinoma patients.

Characteristic	Values
Age, years	
Median	50
Range	30–69
Gender, n (%)	
Male	20 (74.1)
Female	7 (25.9)
Smoking habit	
Smokers	13 (48.2)
Ex-smokers	9 (33.3)
Non-smokers	5 (18.5)
Weight loss, kg	
≤5	25 (92.6)
>5	2 (7.4)
ECOG performance status	
0	17 (63.0)
1	10 (37.0)
Stage, n (%)	
IIIB	4 (14.8)
IV	21 (77.8)
Unknown	2 (7.4)
Histology, n (%)	27 (100)

ECOG, Eastern Cooperative Oncology Group.

**Table II:** Variations of associated tumor biomarkers among the PR, SD and PD subgroups.

Parameters	PR (n=9)	SD (n=9)	PD (n=9)	P-value
CEA (μg/l)	54.60±4.77	232.82±6.13	31.19±3.64	0.295
CA125 (U/ml)	208.97±3.57	295.29±4.55	938.37±12.68	0.368
CA153 (U/ml)	56.43±5.89	29.58±1.55	31.01±2.79	0.181
CA199 (U/ml)	22.02±3.48	527.27±13.30	45.69±7.94	0.372
CA724 (U/ml)	7.31±2.44	15.45±1.92	2.84±0.47	0.281
CYFRA21-1 (ng/ml)	110.19±10.74	6.39±5.35	13.68±15.18	0.414
NSE (ng/ml)	20.63±7.17	22.79±1.08	16.50±2.74	0.696
SCC (ng/ml)	0.98±0.37	3.60±1.44	0.87±0.51	0.492

CEA, carcinoembryonic antigen; CA, carbohydrate antigen; CYFRA21-1, cytokeratin 19 fragment; NSE, neuron-specific enolase; SCC, squamous cell carcinoma; PR, partial response; SD, stable disease; PD, progressive disease.

**Table III:** Spearman’s correlation coefficient between the serum leptin and tumor biomarkers.

Clinical parameters	Correlation coefficient	P-value (2-tailed)
CEA	−0.023	0.885
CA125	−0.025	0.883
CA153	0.007	0.966
CA199	−0.048	0.761
CA724	0.175	0.288
CYFRA21-1	−0.110	0.493
NSE	−0.168	0.308
SCC	−0.398	0.012^a^

CEA, carcinoembryonic antigen; CA, carbohydrate antigen; CYFRA21-1, cytokeratin 19 fragment; NSE, neuron-specific enolase; SCC, squamous cell carcinoma.
